# The exposome paradigm for severe mental disorders: is it useful for clinical practice?

**DOI:** 10.1192/j.eurpsy.2025.253

**Published:** 2025-08-26

**Authors:** S. Guloksuz

**Affiliations:** 1 Psychiatry, Maastricht University Medical Center, Maastricht, Netherlands; 2 Psychiatry, Yale School of Medicine, New Haven, United States

## Abstract

**Abstract:**

In this presentation, I will explore how the exposome paradigm can be leveraged to advance clinical practice in psychiatry. Specifically, I will highlight the potential of cumulative environmental risk scores to predict outcomes in severe mental disorders, such as the exposome score for schizophrenia (ES-SCZ).

Numerous socio-environmental factors have been linked to mental disorders, including childhood adversities, stressful life events, substance use, obstetric complications during pregnancy and childbirth, and urban living. Environmental factors do not exist in isolation; they form complex networks of interrelated and interactive elements. In this regard, the exposome represents the totality of an individual’s environmental exposures throughout their lifetime. The exposome framework introduces a holistic approach to embrace this complexity and a theoretical framework to investigate the poly-gene and poly-environment etiology of psychiatric disorders.

Guided by the exposome framework, we have recently estimated the ES-SCZ, a cumulative environmental exposure score for schizophrenia, including cannabis use, winter-birth, hearing impairment, bullying, and five domains of childhood adversities (emotional and physical neglect, along with emotional, sexual, and physical abuse). The ES-SCZ successfully differentiated individuals with schizophrenia, accounting for 28% of the variance in an independent case-control sample. Subsequently, we have tested the risk stratification properties and the predictive performance of the exposome score for schizophrenia in the general population. The ES-SCZ had strong discriminative performance for schizophrenia (AUC = 0.84) and was associated with the degree of psychosis risk in the general population. Finally, we tested the performance of ES-SCZ for dissecting the functional and symptomatic outcome heterogeneity in patients with psychosis in four different cohorts (EUGEI, GROUP, Athens FEP, and HAMLETT-OPHELIA). ES-SCZ was associated with poor overall functioning and cognitive impairment at baseline and follow-up visits. ES-SCZ was also temporally associated with poor symptomatic improvement from baseline to follow-up assessments, particularly the negative symptom dimension. Furthermore, models that included the polygenic risk score for schizophrenia and clinical features showed that the relationship between ES-SCZ and functional outcomes cannot be explained by genetic or clinical risk factors alone.

Overall, our findings demonstrate the potential benefits of the exposome score for schizophrenia, which can be integrated for early detection, outcome prognostication, clinical staging, and risk stratification in the future.

**Disclosure of Interest:**

None Declared

